# Choosy Moral Punishers

**DOI:** 10.1371/journal.pone.0039002

**Published:** 2012-06-13

**Authors:** Christine Clavien, Colby J. Tanner, Fabrice Clément, Michel Chapuisat

**Affiliations:** 1 Department of Ecology and Evolution, University of Lausanne, Lausanne, Switzerland; 2 Cognitive Science Centre, University of Neuchâtel, Neuchâtel, Switzerland; Hungarian Academy of Sciences, Hungary

## Abstract

The punishment of social misconduct is a powerful mechanism for stabilizing high levels of cooperation among unrelated individuals. It is regularly assumed that humans have a universal disposition to punish social norm violators, which is sometimes labelled “universal structure of human morality” or “pure aversion to social betrayal”. Here we present evidence that, contrary to this hypothesis, the propensity to punish a moral norm violator varies among participants with different career trajectories. In anonymous real-life conditions, future teachers punished a talented but immoral young violinist: they voted against her in an important music competition when they had been informed of her previous blatant misconduct toward fellow violin students. In contrast, future police officers and high school students did not punish. This variation among socio-professional categories indicates that the punishment of norm violators is not entirely explained by an aversion to social betrayal. We suggest that context specificity plays an important role in normative behaviour; people seem inclined to enforce social norms only in situations that are familiar, relevant for their social category, and possibly strategically advantageous.

## Introduction

Social norms are key to human cooperative interactions [1], [2]. When efficiently enforced by punishment, they usually help to restrain free-riding behaviour in large groups of unrelated individuals [2], [3], [4], [5], [6]–but see [7], [8]. It is not clear however, what factors drive people to punish norm violators. Depending on the situation, there are various types of motivations for punishing social norm violators, such as the drive to increase personal reputation in a social context [9], [10] or the urge to retaliate after having been victim of anti-social behaviour [11]. One further possible motivation that has raised much interest in the literature–because it directly enforces social norms–is the aversion to social betrayal [12], [13], [14], [15]. Our study focuses on this particular type of motivation.

Studies making use of economic games reveal a human disposition to spend money to punish social norm violators even while knowing that no further round of the game will be played–therefore no long term monetary reward from future cooperation can be expected [16], [17], [18]. This propensity to enforce social norms has been observed in third-party situations where subjects witness an interaction in which they are not personally involved, are prompted to consider the behaviour of one actor as wrong, and have the possibility–but no obligation–to punish the wrongdoer [12], [19]. To a lesser extent, third-party punishment can also be observed when anonymous test conditions are secured–thus, no gain through reputation can be expected from applying punishment [10], [20]_ENREF_17.

These experimental results have led some experimental economists and evolutionary anthropologists to propose that humans have evolved a strong and universal psychological disposition to punish norm violators [12], [13], [14], [15], [21]_ENREF_6_ENREF_7. This disposition–sometimes called “universal structure of human morality” [12] or “pure aversion to social betrayal” [13]–would be triggered when somebody reveals an anti-social intention by violating a group-beneficial norm [14]. This aversion to social betrayal is considered to be present in all cultures, although important individual differences in its expression are acknowledged–depending on individual personality types, this aversion can be expressed more or less vividly. Culture-specific patterns of norm enforcement–which norm violations are likely to be sanctioned–are attributed to cultural evolution, which shapes the specific content of the norms that are enforced in a given population [15], [22]. Thus, this assumption of a “pure aversion to social betrayal” does not imply that all humans always punish norm violations or that humans sanction the same norm violations in all cultures; it predicts however that a significant proportion of any sufficiently large group of humans will punish violations of local norms.

The occurrence and generality of such aversion to social betrayal is an important topic because it is tightly connected with the cross-disciplinary debate over the extent to which people are altruistically motivated to help others or contribute to the common good [23], [24], [25]. However, the scope of current results on third-party punishment remains difficult to evaluate because they have mainly been obtained in laboratory test conditions [26], [27], with the use of monetary games that allow for testing only specific norms–equity and equality norms. Moreover, in these games, participants might easily misunderstand the instructions [28] or alter their behaviour when they are aware of being studied or when they have taken similar tests before [29]. Finally, research in anthropology and sociology cast doubt on the idea that, in the real world, people are willing to sanction others' social norm violations if there is no social pressure or personal interest at stake for a review, see [27].

**Figure 1:**
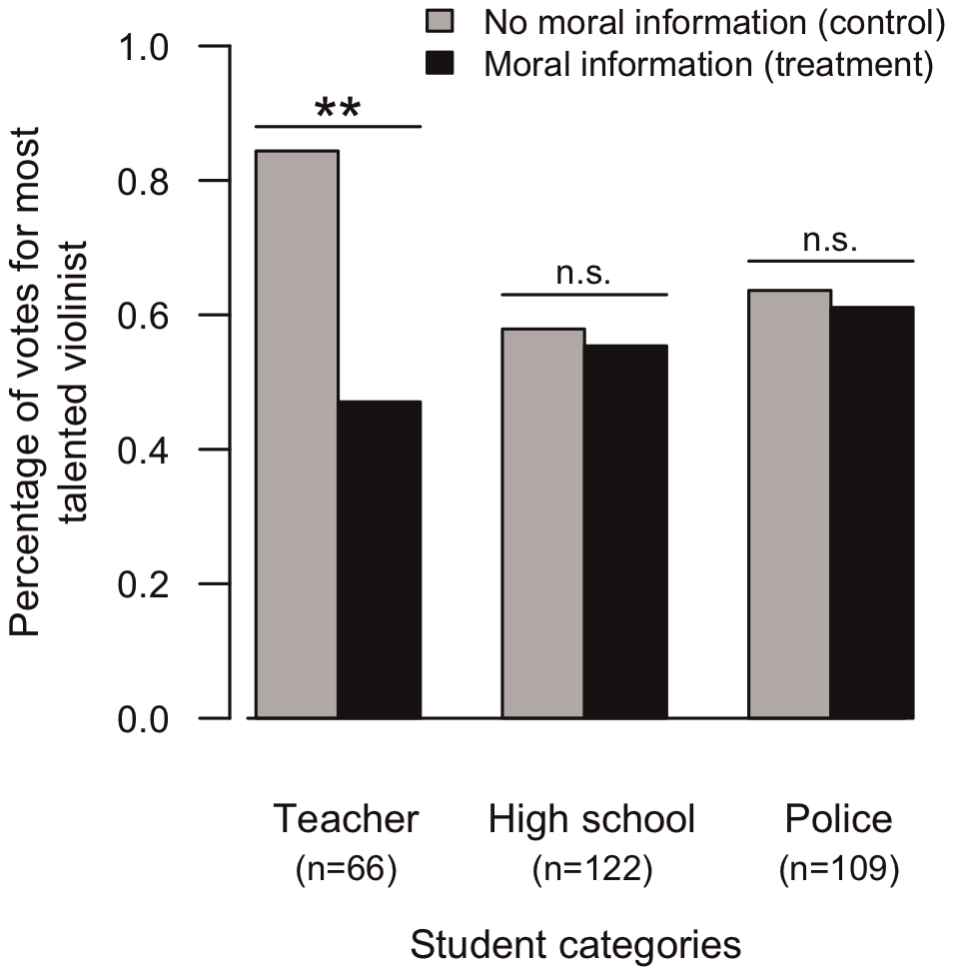
Proportions of votes for the most talented violinist among teacher, high school and police students when they received the moral information–i.e. she is immoral–(black bars) or when they received the neutral information–i.e. she is socially integrated–(grey bars). Significance levels (mixed-effect GLM): ** = p<0.01, n.s. = p>0.05.

Interestingly this debate parallels an important and old standing controversy among philosophers. Proponents of the “internalist” view contend that moral judgments are necessarily motivating [30], [31], whereas advocates of the “externalist” view think that the source of motivation lies outside moral judgments–e.g. social pressure or personal interest [32], [33]. Internalists would endorse the idea of pure aversion to social betrayal, whereas externalist would reject it.

To challenge the idea of a universal propensity to punish social betrayal, and to contribute experimentally to the philosophical debate, we conducted a real-life, non-economic, third-party study implicating different socio-professional categories. We used a novel exploratory approach that avoids confounding factors such as reputation, retaliation, or monetary incentive, while testing participants who do not expect their moral behaviour to be studied. In an everyday school context, while not knowing that they were being studied, student-participants have been invited to vote for young violinists in a music competition staged on video. The participants were also informed that one of the violinists had a reputation for moral misconduct because she had repeatedly attempted to ruin the quality of others' work. Participants had no formal obligation to intervene, but they could punish the immoral violinist by diminishing her career opportunities. The decision to punish had no impact on participants' lives: the wrongdoer was not expected to be encountered in the future, and punishment provided no reputation because it was done anonymously. We tested participants from the same territorial and socio-cultural area who could agree on the violinist's immoral character. Participants differed, however, in their career trajectories: they were future teachers, future police officers, and high school students.

Our aim was to find out whether, and to what extent, people are inclined to apply non-self-interested–i.e. not motivated by expectation of future rewards such as reputation–punishment against a moral norm violator in a real-life context. We evaluated the universal character of people's propensity to punish social betrayal by examining three categories of participants with similar cultural backgrounds. If cost-free third-party moral punishment is not applied equally in the three categories despite shared moral disapproval, this would cast doubt on the “pure aversion to social betrayal” hypothesis, and call for further investigations of the motivational factors responsible for moral punishment.

## Materials and Methods

### Ethics Statement

This study has been approved by our local ethics committee: “Comission cantonale d'éthique de la recherche sur l'être humain”, University of Lausanne. Throughout the whole test procedure, participants were unaware of being studied, thus, they were not asked for written informed consent. Instead, we organised post-experimental debriefing sessions during which we asked for verbal consent. We took note that none of the participants expressed discomfort or asked to withdraw their data from the study after having been informed of their involvement in a scientific experiment. Votes and questionnaires were completed anonymously: neither fellow participants nor experimenters could know individual responses. The experimenters and the ethics committee considered the above described test procedure as adequate because, from participants' perspective, the study is not more invasive than an anonymous opinion survey.

### Methods

During ordinary class lessons, we simulated the final phase of a violin competition: representatives of a music company – in fact experimenters – entered in student-participants' classrooms and asked them to act as music judges in the final phase of a violin competition (see File S1, section 1 for detailed test procedure and materials). Participants were students from three different types of schools: preparatory for becoming teachers (n = 66, 3 classes from 2 different schools, age 18–35), advanced high school (n = 122, 7 classes from 2 schools, age 14–18), and preparatory for becoming police officers (n = 109, 1 class, age 19–43).

Participants sat at a computer with headphones, heard a professional recording of an excerpt from a Mozart Violin Concerto, followed by filmed recordings of the two pre-selected violinists playing the same piece. Both violinists were professional female performers of similar appearance, whose faces could not clearly be seen, and both were described as finishing students from a European music school. Participants also received additional information from short interviews of the violinists' former music professor: the professor provided “technical information”–e.g. she usually is very right in her tone–and “social information”–e.g. she has always been well integrated in her class. Participants were told that the winning violinist would be awarded a record deal, which represents a significant career improvement.

By design, one violinist's musical performance was better–according to professional standards–than the other; we used respectively the best and the worst version of a series of pre-recorded performances by each musician. Control and treatment conditions differed with respect to the social information provided by the professor. Participants in the control condition (teacher, n = 32; high school, n = 57; police, n  = 55) received equally positive information regarding both violinists' social character. In the treatment condition (teacher, n = 34; high school, n = 65; police, n  = 54), however, the less talented violinist was described in a socially positive way, whereas the more talented violinist was described as morally disrespectful: she showed repeated moral misconduct such as mistuning fellow students' instruments or mixing their musical scores just before concerts. Also, because the order in which information is provided can influence people's choices [34], [35], we randomized the order in which participants viewed violinists in both the control and treatment conditions.

After observing the musical performances and professor interviews, participants voted for one violinist that they considered worthy of career advancement, followed by a short questionnaire in which they reported their gender, age, and interest in classical music. In most experiments (3 teacher classes, n = 66; 3 high school classes, n = 36; the large police class, n  =  109), participants were also asked what factors played a role in their voting decision–e.g. technical considerations, feelings, moral considerations. Post-experimental debriefing sessions confirmed that participants believed the cover story–except for 12 sceptical participants that were discarded from the analysis–and were convinced that their decisions had real repercussions.

### Statistical Analysis

To test whether socio-professional categories differed in their voting behaviour, we analysed the full dataset with a generalized linear mixed-effects model (GLM, family = binomial) [36], including the following fixed factors and their interactions: gender, interest in classical music, socio-professional category (teacher, high school and police), moral information about the most talented violinist's behaviour (socially integrated versus immoral), violinist viewing order, and the residuals of age category (≤18, >18) regressed on socio-professional category–we used residuals here because age and socio-professional category were positively collinear (r = 0.171, p = 0.003) [37]. To account for variation among classes–because teacher and high school categories were composed of two schools each divided into several classes–, we assigned class as a random effect. We sequentially simplified the full model (significance criterion: p<0.05) by removing non-significant effects beginning with highest-order interactions until we obtained a final model. Because the three socio-professional categories differed with respect to the propensity to punish, we repeated the same procedure for each category (teacher, high school and police) separately.

To determine if the three socio-professional categories differed in their stated receptivity to moral information, we analysed the written information left by participants in the treatment condition (data from 104 participants). With a two-sided Fisher's exact test, we compared the number of participants from each socio-professional category who stated that moral information–solely or among other criteria–influenced their vote, to the number of participants that did not mention moral information as a factor in their decision. In a post hoc analysis, we also used a one-sided Fisher's exact test to determine whether participants' stated receptivity to moral information was correlated with the order in which they viewed violinists.

## Results

**Figure 2:**
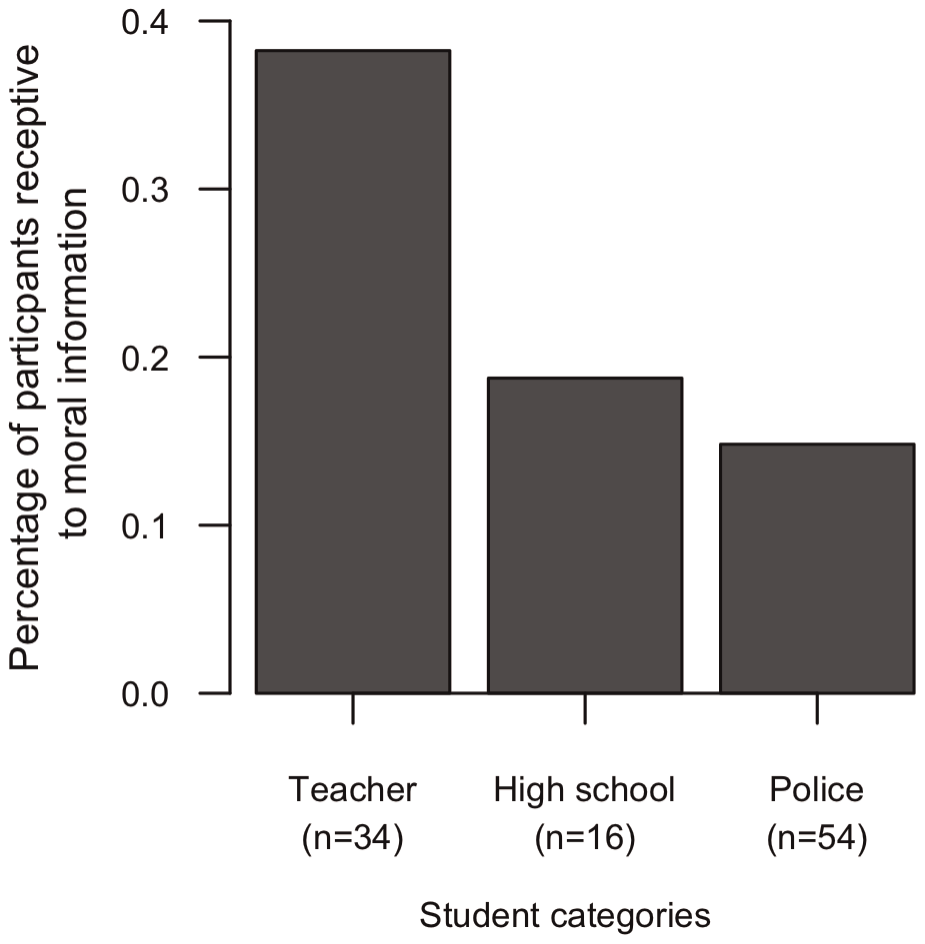
Proportion of participants exposed to moral information reporting that moral information influenced their decision. Overall proportions differed significantly among socio-professional categories (two-sided Fisher's exact test: p = 0.048).

The propensity to punish social misconduct–i.e. to vote against the immoral violinist despite the fact that she was more talented–varied among participants with different career trajectories (mixed-effect GLM: interactions between moral information and socio-professional categories: high school and teacher categories z = 2.15, p = 0.031, police and teacher categories z = 2.12, p = 0.034; Table 1). Future teachers punished the most talented violinist according to her past social misconduct, with 37% less votes for her when she was described as immoral (mixed-effect GLM: z = −3.10, p = 0.002; Fig. 1). In contrast, future police officers and high school students did not show significant third-party moral punishment, with only a 2.5% decrease in votes in response to social misconduct, on average (mixed-effect GLM: police students: z = −0.553, p = 0.580; high school students: z = −0.802, p = 0.423; Fig. 1). Consistent with this outcome, future teachers reported significantly more receptivity to moral information–immoral character of the violinist–than did future police officers and high school students (Fisher's exact test: p = 0.048; Fig. 2). Specifically, 38% of the future teachers stated that moral information influenced their vote, in contrast to 19% and 15% of the high school and police students, respectively. None of these participants voted for the immoral violinist.

**Table 1:** Summary of the final generalized mixed-effects model including participants from all three socio-professional categories (dependent variable = vote for or against the most talented violinist; reference category = teacher).

Factor	coefficient	z value	*p*
Moral information	−2.01	−3.03	0.002
Violinist viewing order	−2.03	−7.05	< 0.001
Interest in classical music	1.33	3.58	< 0.001
Police vs. Teacher categories	−0.99	−1.61	0.108
High school vs. Teacher categories	−1.42	−2.33	0.020
Moral info * Police vs. Teacher categories	1.69	2.12	0.034
Moral info * High school vs. Teacher categories	1.69	2.15	0.031

The two last lines in the table report significant interactions between how participants responded to moral information (voted for or against the immoral versus socially integrated violinist) and which socio-professional category they belonged to (comparing either police or high school to teacher). This indicates that both police and high school categories differed from the teacher category with respect to their propensity to punish. Moreover, the order in which participants viewed the violinists, as well as participants' stated interest in classical music, were significant.

The viewing order affected participants' judgment overall (mixed-effect GLM: z = −7.05, p<0.001; Table 1) and in each category of participants (high school: z = −5.06, p<0.001; police: z = −4.25, p<0.001; teacher: z = −2.46, p = 0.014). In all cases, more votes went to the violinist that was presented last (File S1, section 2.1). Moreover, self-reported interest in classical music was associated with a significant increase in votes for the most talented violinist, both overall (mixed-effect GLM: z = 3.58, p<0.001; Table 1) and in the high school category (z = 2.64, p = 0.008).

The teacher category was better at identifying the most talented violinist (this was expected notably because preparatory schools for teachers provide more music training than police and high schools), which induces a difference among categories in our control condition (Fig. 1). There is no plausible relationship between this difference and the effect of moral information. Moral information clearly influenced teachers' decisions. Had police and high school students been equally responsive to moral information, they should have punished the immoral violinist even more than teachers, as the violinist's misbehaviour was the only relevant discriminating information they received, given that they were not as good at judging the music. But this was not how the future police and high school students behaved.

Lastly, there was a correlation between acknowledged receptivity to moral information and viewing order (Fisher's exact test: p = 0.025). Significantly more participants who reported being influenced by the moral information had seen the immoral violinist first. As the viewing order biases the votes in favour of the violinist presented last, it suggests that participants were more receptive to moral information when it was compatible with a choice of action–vote for the last violinist–they were already inclined to make. The extensively studied tendency of post-hoc rationalisation for one's decisions can easily account for this result [38], [39] (more details in File S1, section 2.2).

## Discussion

We found limited evidence for non-self-interested moral punishment. Future teachers punished the immoral violinist although they were not personally affected by her wrongdoing, had no formal obligation to sanction, and could not expect a benefit through reputation or future cooperation from punishing. This propensity to punish the norm violator, however, varied greatly among socio-professional categories. In sharp contrast with future teachers' behaviour, police and high school students did not sanction the immoral violinist. This difference appears clearly in participants' voting decisions (Fig. 1), as well as their expressed statements about what influenced their decisions (Fig. 2). Note that the lack of motivation to punish is particularly striking in light of the fact that the decision to punish did not imply any form of cost–neither monetary nor reputational–for the punisher.

In numerous writings, experimental economists and evolutionary anthropologists have taken the observation of third-party punishment as evidence for the existence of a simple universal punishing mechanism [12], [13], [14], [15], [21]. Our results challenge this broad assumption in revealing behavioural variations across different categories of participants who could all acknowledge the obviously immoral character of the violinist–she showed basic disrespect towards her fellow students in mistuning their instruments or mixing their musical scores just before concerts. Future teachers punished, but not police and high school students. This suggests that people are not genuinely motivated to enforce the general social norms to which they abide. Additional conditions to the bare recognition of social misconduct seem to be needed before individuals are motivated to apply punishment.

Most variation previously observed in people's propensity to apply third-party punishment has been compatible with the idea of a universal aversion to social betrayal. For example, the substantial differences reported across societies [5], [22], [40] might reflect the moral standards particular to cultures, where some cultures are more receptive to a given norm than others [5], [8], [15]. Similarly, the important differences across studies conducted in western societies–third-party punishment can vary from 10% [19], [20] to 60% [12]–could be explained by differences in experimental designs. For example, motivation to inflict third-party punishment can be enhanced or disrupted by subtle cues that refer to external factors such as reputation [10], [21], expectation of future cooperation [19], or monetary incentives [41]. The variation observed in our experiment, however, resists these explanations. Indeed, all participants shared the same broad socio-cultural background and were exposed to an identical test protocol in which neither reputation nor future cooperation nor monetary gains could be expected–because votes and questionnaires were completed anonymously.

There is room for speculation about the reasons why only future teachers enforced the moral norm. First, one might think that only teachers understood the violinist's behaviour as immoral. This hypothesis is not very convincing though, as an important number of participants in the police (39%) and high school (44%) categories explicitly mentioned her misbehaviour in the questionnaire–most of them stated that they were aware of the immoral character of the violinist but decided to discard this information. Second, the three categories may have had a different understanding of the task that was assigned to them. However, the comments left by participants did not reveal any marked difference in their understanding of the task–i.e. vote anonymously for the candidate they wanted to promote. Third, there might be selection for certain personality types within certain career paths. For example, individuals who are more sensitive to norm violation may be more likely to become teachers. However, in that case, some non-negligible proportion of punishers should also be present in the high school category, as teachers–but not police officers–need to complete high school before starting their preparatory school.

Some elements relevant to understanding teachers' behaviour are worth emphasising. First, their social role is to educate students and the cover story presents a teacher referring to the moral misbehaviour of one of his music students in a school context. Thus, this type of norm violation may be more relevant for teachers. In contrast, the main role of police officers is to enforce state laws, rather than socially accepted moral norms. Second, it is strategically advantageous for teachers to be able to punish undisciplined students so as to discourage them from disturbing the class atmosphere in the future. These facts might indicate that people are more inclined to enforce social norms in familiar contexts with clear assignments of social duties, and among these contexts, possibly those in which widespread obedience to the social norm provides benefits to their social category. If the strategic aspect plays a crucial motivational role in punishment, externalist views of moral motivation would gain empirical support.

Our results are consistent with psychology and economics literature that reveal the context-sensitive aspects of human normative behaviour [42], [43]. For example, people show increased norm obedience when their attention has been previously drawn to the norm or to a closely related norm [42], or when they expect most of their neighbours to follow the norm [43]. The idea of stable norms of cooperation has also been recently challenged with results showing that population size affects individuals' propensity to cooperate [44]. More directly relevant to third-party punishment behaviour, two economic experiments point to the importance of the strategic aspects of normative situations: the propensity to apply third-party punishment declines when out-group members–as opposed to in-group members–have been a victim of wrongdoing [45] or when participants are informed of the presence of a second third-party that can punish the same wrongdoer [20]._ENREF_18_ENREF_18_ENREF_18_ENREF_18 Parochialism and diluted responsibility are strategic factors that seem to impact directly on people's receptivity to social norm violation and motivation to apply punishment.

To sum up, people turn out to be choosy with respect to the norms they are willing to enforce in particular circumstances. The mismatch between people's evaluation of a norm violation and their willingness to enforce the moral norm shows that other causal factors than a pure aversion to social betrayal are operating. Familiarity of the context in which the norm is violated, and duties associated with social functions might play a role. They are possibly coupled with sensitivity to strategic aspects of the social context. Actors might not be consciously aware of such sensitivity though, leaving open the question as to what extent the resulting behaviour counts as moral. Further studies will help identify more precisely whether, and to what extent, these factors are needed to elicit punishment. For example, it would be interesting to manipulate independently the social and the strategic relevance of the norm for various socio-professional categories.

## Supporting Information

File S1 **Test procedure and material and supplementary statistics.**
(PDF)

## References

[1] Bowles S (2004). Microeconomics: behavior, institutions, and evolution.. New York: Princeton University Press.

[2] Ostrom E (1990). Governing the commons: The evolution of institutions for collective action.. Cambridge: Cambridge University Press.

[3] Bowles S, Gintis H (2004). The evolution of strong reciprocity: cooperation in heterogeneous populations.. Theoretical Population Biology.

[4] Fehr E, Gächter S (2002). Altruistic punishment in humans.. Nature.

[5] Henrich J, Ensminger J, McElreath R, Barr A, Barrett C (2010). Markets, Religion, Community Size, and the Evolution of Fairness and Punishment.. Science.

[6] Chaudhuri A (2011). Sustaining cooperation in laboratory public goods experiments: a selective survey of the literature.. Experimental Economics.

[7] Gachter S, Herrmann B (2011). The limits of self-governance when cooperators get punished: Experimental evidence from urban and rural Russia.. European Economic Review.

[8] Herrmann B, Thoni C, Gachter S (2008). Antisocial punishment across societies.. Science.

[9] Barclay P (2006). Reputational benefits for altruistic punishment.. Evolution and Human Behavior.

[10] Kurzban R, DeScioli P, O'Brien E (2007). Audience effects on moralistic punishment.. Evolution and Human Behavior.

[11] de Quervain DJF, Fischbacher U, Treyer V, Schellhammer M, Schnyder U (2004). The neural basis of altruistic punishment.. Science.

[12] Fehr E, Fischbacher U (2004). Third-party punishment and social norms.. Evolution and Human Behavior.

[13] Fehr E, Camerer C (2007). Social neuroeconornics: the neural circuitry of social preferences.. Trends in Cognitive Sciences.

[14] Carpenter JP, Matthews PH, Ong'ong'a O (2004). Why punish? Social reciprocity and the enforcement of prosocial norms.. Journal of Evolutionary Economics.

[15] Gintis H, Henrich J, Bowles S, Boyd R, Fehr E (2008). Strong reciprocity and the roots of human morality.. Social Justice Research.

[16] Gintis H (2000). Strong Reciprocity and Human Sociality.. Journal of Theoretical Biology.

[17] Fehr E, Fischbacher U (2003). The nature of human altruism.. Nature.

[18] Carpenter JP (2007). The demand for punishment.. Journal of Economic Behavior & Organization.

[19] Carpenter JP, Matthews PH (2012). Norm enforcement: anger, indignation, or reciprocity?. Journal of Economic Behavior & Organization.

[20] Lewisch PG, Ottone S, Ponzano F (2011). Free-Riding on Altruistic Punishment? Experimental Comparison of Third-Party-Punishment in a Stand-Alone and in an In-Group Environment.. Review of Law & Economics.

[21] Rockenbach B, Milinski M (2006). The efficient interaction of indirect reciprocity and costly punishment.. Nature.

[22] Henrich J, McElreath R, Barr A, Ensminger J, Barrett C (2006). Costly punishment across human societies.. Science.

[23] Batson CD (1991). The altruism question: Toward a social psychological answer.. Hillsdale: Lawrence Erlbaum.

[24] Stich SP, Doris JM, Roedder E, Doris JM (2010). Altruism..

[25] Clavien C, Klein RA (2010). Eager for fairness or for revenge? Psychological altruism in economics.. Economics and Philosophy.

[26] Gigerenzer G (2000). Adaptive thinking: rationality in the real world.. New York: Oxford University Press.

[27] Guala F (2012). Reciprocity: Weak or strong? What punishment experiments do (and do not) demonstrate.. Behavioral and Brain Sciences.

[28] Houser D, Kurzban R (2002). Revisiting kindness and confusion in public goods experiments.. American Economic Review.

[29] Pelham BW, Blanton H (2007). Conducting research in psychology: measuring the weight of smoke.. Australia; Belmont, CA: Thomson Wadsworth.

[30] Brink DO (1989). Moral realism and the foundations of ethics.. Cambridge, New York: Cambridge University Press.

[31] Dancy J (2000). Practical reality.. Oxford; New York: Oxford University Press.

[32] Svavarsdóttir S (1999). Moral cognitivism and motivation.. Philosophical Review.

[33] Copp D (1997). Belief, reason, and motivation: Michael Smith's The Moral Problem.. Ethics.

[34] Bornstein RF (1989). Exposure and Affect: Overview and Meta-Analysis of Research, 1968–1987.. Psychological Bulletin.

[35] Pandelaere M, Millet K, Van den Bergh B (2010). Madonna or Don McLean? The effect of order of exposure on relative liking.. Journal of Consumer Psychology.

[36] Bates D, Maechler M (2009). lme4: Linear mixed-effects models using S4 classes.. R package version 0999375–32.

[37] Graham MH (2003). Confronting multicollinearity in ecological mutliple regression.. Ecology.

[38] Haidt J (2001). The emotional dog and its rational tail: A social intuitionist approach to moral judgment.. Psychological Review.

[39] Festinger L (1957). A theory of cognitive dissonance.. Evanston: Row, Peterson.

[40] Marlowe FW (2009). Hadza Cooperation–Second-Party Punishment, Yes; Third-Party Punishment, No. Human Nature.

[41] Ottone S (2008). Are people Samaritans or Avengers?. Economics Bulletin.

[42] Kallgren CA, Reno RR, Cialdini RB (2000). A focus theory of normative conduct: When norms do and do not affect behavior.. Personality and Social Psychology Bulletin 26.

[43] Bicchieri C, Xiao E (2009). Do the Right Thing: But Only if Others Do So.. Journal of Behavioral Decision Making.

[44] Lamba S, Mace R (2011). Demography and ecology drive variation in cooperation across human populations.. Proceedings of the National Academy of Sciences.

[45] Bernhard H, Fischbacher U, Fehr E (2006). Parochial altruism in humans.. Nature.

