# Supplementary material for: Choosy Moral Punishers
Source: PLoS One. 2012 Jun 13;7(6):e39002. doi: 10.1371/journal.pone.0039002 (PMC3374788; doi:10.1371/journal.pone.0039002)
Supplement: File S1 — Test procedure and material and supplementary statistics. (PDF) [file pone.0039002.s001.pdf]

File S1 - Supporting information

Choosy moral punishers

Christine Clavien, Colby J. Tanner, Fabrice Clément and Michel Chapuisat

Table of content

1. TEST PROCEDURE AND MATERIAL 1
1.1. Recruitment and general features of test procedure 1
1.2. Description of the cover story, as presented to the participants 2
1.3. Translation of professor's comments 5
1.4. Translation of the questionnaire 6
2. SUPPLEMENTARY STATISTICS 9
2.1. Viewing order effect 9
2.2. Correlation between viewing order and acknowledged influence of moral information 10
2.3. Factors that were not significant 11

1. TEST PROCEDURE AND MATERIAL

1.1. Recruitment and general features of test procedure

We made contact with different schools to obtain the authorization to conduct our study in one or several of their classes. In each school, we organized one test day. Participants were not informed of the study. They were only asked to take headphones and come to the computer room of their school during one of their ordinary lesson (if possible, we choose a music lesson). If more than one class was being tested, we made sure that students from the different classes would not meet during the breaks.

On the test day, representatives of the music company *Euroclassic Records*—the music company was in fact fictitious and the representatives were the experimenters—presented the cover story to the participants and oversaw the completion of the experiment. Participants were asked to intervene as music judges in a music competition organized by the company. The judges task was to a) watch and hear a professional recording of an excerpt from a Mozart Violin Concerto, followed by video recordings of two pre-selected violinists playing the same piece, followed by an interview of the violinists' former music professor (see translation of professor's comments), b) vote for one out of the two violin players, and c) respond to a few general questions (see translation of the questionnaire). Votes and questionnaires were

completed anonymously on computers, saved on unlabelled USB sticks and stored in a common bag—thus, even experimenters could not know the decisions of the participants. This procedure lasted for about 45 minutes. In a post-experiment debriefing session, participants were informed that they had in fact been involved in a scientific experiment. None of them expressed discomfort or asked to withdraw her/his data from the study. Moreover, debriefing sessions confirmed that participants believed the cover story and were convinced that their decisions had real repercussions. There were a few exceptions: we discarded from the analysis all sceptical participants (12 in total) who expressed their doubts during the test or while handing back the USB stick or who wrote explicit critical commentaries in the questionnaire.

1.2. Description of the cover story, as presented to the participants

Hello, I'm a representative of the new record company called *Euroclassic Records*. More precisely, I'm an external consultant whose job is to organize the music competition in which today you are going to be a member of the jury.

My assistants will help you with any technical problems you encounter during the competition and subsequent voting procedure.

Euroclassic *Records* is a new music company, founded at the beginning of this year, and is in a launching phase for new music releases.

- The Belgian benefactor, Heinrich VonSattle (1940-2009), has bequeathed an important sum of money to help launch the company.
- During his life, Mr. VonSattle made his fortune in the import/export industry, but in his private life he was a connoisseur of classical music. One of his major regrets was that European classical music was not currently well represented at the international level.
- For your information, the classical music industry is currently dominated by China and Russia. These countries provide artists who have a remarkable technical mastery. This is partly due to the teaching system; at very young age, the children who show talent are trained full time in music. In comparison, in Europe, talented children need to follow a traditional curriculum in addition to musical training.
- The wish of Mr. VonSattle was to provide more visibility to the European classical music scene. He also wanted to give the opportunity to young European talent to establish musical careers.
- For these reasons, he left part of his fortune to create a foundation dedicated to European classical music. This foundation has capitalized and finances our company, *Euroclassic Records*.

Currently we are preparing an advertising campaign for the company's launch. To begin with, *Euroclassic Records* is producing three products.

- The *Classics* collection: well-known musicians playing sonatas and chamber music. Three CDs have already been recorded with the three well-known artists:
    - Jörg Widmann (German clarinettist)
    - Susanna Mälkki (Finnish cellist)
    - Shani Diluka (French-Sri Lankan pianist). Although technically she is from Sri Lanka, as she grew up in France we consider her European.
- The *Live* collection: this summer, our record company recorded live concerts in three of the most important European classical music festivals:
    - Wiener Festwochen (Vienna – May & June)
    - Festival Chopin (Paris, at the Orangerie de Batatelle – June & July)
    - Lucerne Summer Festival (Lucerne – August & September)
- The *New talents* collection: (including students from top European music schools).

How New talents is organized:

- *Euroclassic Records* has organized a series of competitions in 10 top music schools from different European countries. The goal is to produce and release one new CD composed of solos played by new artists selected from these competitions.
- To ensure objectivity, as well as producing a product with public appeal that is marketable on a large scale, the company has dictated specific rules for this competition (see scheme with visual description of this procedure).
- A filmed competition is organized for each participating school. Participants play the same piece of music (a cadence from Mozart: end of first movement of concerto for violin number 4 in D major K. 218).
- In the first phase of the selection procedure, a professional jury views these filmed performances and selects two candidates from each school.
- To ensure that contest winners appeal to the public, the second and final phase of the selection procedure entails ten groups of non-professional juries.
    - Each group of juries has the task of choosing the best candidate in one school out of the two candidates that had been chosen during the first phase.
    - In this stage, the jury sees the filmed performances and receives supplementary information about the candidates from interviews of one of their former music professors.
    - Judges then vote anonymously because the music company wants to ensure that judges do not feel influenced in any way.
- The ten candidates passing the second selection stage will decide with *Euroclassic Records* which solo piece they will play on the CD.

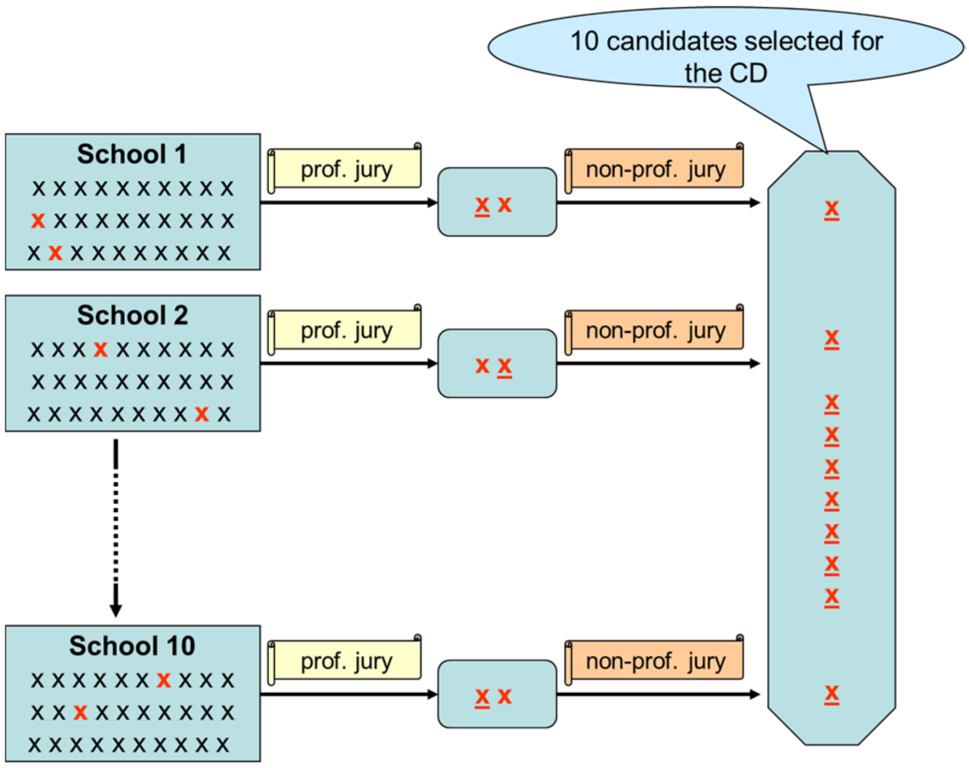

Visual description of the competition procedure (projected to participants)

Euroclassic Records takes on all costs of the competition. It pays the professional jury and it offers to the non-professional jury members one copy of the final CD, produced at the end of all competitions.

- Those of you who desire to receive this CD, please leave your name and address on the paper that will be passed around. We will send a CD to each address on this sheet. It has no connection with the voting procedure, which will be anonymously completed on computer.

Technical information:

- For your information, not all of you will see the same candidate first. So it does not make sense to try to look at whom your neighbour is voting for. Just concentrate on your own evaluation.
- After the vote, we will ask you to respond to a few general questions. The information you will provide are useful for our music company; it helps us better grasp current musical tastes.
- If you have any questions at any time during the process, please raise your hand and my assistants will help you.
- When you are finished, please save your file and give your unlabelled USB stick to one of the assistants. We will store all the sticks in a common bag. Once this is done, you can leave the room and return to your classroom.

1.3. Translation of professor's comments

Social Information:

- Morally correct violinist: "She is very much appreciated by everyone. She is well integrated. She is very nice to be around. Everybody likes her; she's in the centre of the social scene."
- Immoral violinist: "With her we had some difficulties. She plays very well, as you could notice, but she poses some difficulties with her behaviour. I am not the only one to think this way; my other colleagues think the same, even the secretary. We regularly receive complaints from her schoolmates. Most of the time it is not really bad, but it's mean. Sometimes she crosses the line. I remember, for example, she mixed the musical scores of one of her colleagues just before a concert, which destabilized the musician that was the victim of this "machination". This disturbed the whole orchestra, and it was really not appropriate. Another of her acts was to mistune the instrument of her neighbour. Well, mistuning an instrument is not so bad, because we can re-tune it very quickly. But it puts the whole group under stress. Overall this person has disappointed us a lot, but I have to say that she is really talented. She has an excellent sound, but there is this aspect to her..."

Technical information:

- Type 1: "This person has a richness in her way of playing and plays with much colour. This makes her music very pleasant to listen to. Moreover, she is very sensible. She is able to make her instrument cry and make the audience feel what she feels while playing. She has a warm and beautiful sound. If I had to say something negative about her way of playing, I would say she sometimes shows stress and stage fright, which hinders the message she wants to convey. But this can be fixed when she has more experience. I have no worry about her future career."
- Type 2: "She has a good, professional technique, and a remarkable level of playing, which all the professors in the school agree with. She handles the instrument in a very good manner. She is good at interpreting and has extraordinary colours in her playing. She handles the bow in a very stable manner. Sometimes while interpreting some pieces of music, her way of playing lacks a little bit of substance. But she is young; this will become better with time."

1.4. Translation of the questionnaire

EUROCLASSIC RECORDS

Euroclassic Records Competition

We are grateful to you for accepting our invition to be part of the jury for this violin competition organized by *Euroclassic Records*.
Be assured that this procedure guarantees judges' anonymity: there is no way to attach your personal identity with the information that you provide in this questionnaire.

Competition procedure: please follow the instruction in the order in which they are given in the document below.

Music

Listen to the three following interpretations of a Mozart cadence (end of the 1st movement from the Concerto for Violin Number 4 in D major K. 218). Each one lasts approximately 2 minutes.

To familiarize yourself with Mozart's cadence, begin by listening to the interpretation by Franco Gulli, one of most well-known violinists of the 20th century, and who died in 2001 (click the button to open the video).

Franco Gulli

Now, listen carefully to the two following interpretations that have been selected by an earlier music jury.

Candidate 1 Candidate 2

Interview

Now, listen to the point of view of the Professor of these two candidates that you have just seen.

Intro interview | About candidate 1 | About candidate 2 | End interview

If you want, you have the possibility to see again each video before moving ahead to the voting procedure.

Vote

For which candidate do you vote? (click one of the corresponding boxes)

☐ I vote for candidate 1

☐ I vote for candidate 2

General Questions

Thank you for responding to the following general interest questions.
(Check the corresponding boxes)

Before today, have you heard of the *Euroclassic Records* company?

☐ *yes*
☐ *no*

Have you chosen to receive the CD that will be recorded with the winner of this competition by *Euroclassic Records*?

☐ *yes*
☐ *no*

On average, how much time per day do you spend listening to music (ipod, CD, radio, etc.) ?

☐ *0 min.*
☐ *from 0 to 30 min.*
☐ *from 30 to 60 min*
☐ *more than 60 min*

Which style(s) of music do you listen to regularly (multiple responses are possible)?

☐ *classic*
☐ *jazz*
☐ *blues*
☐ *traditional*
☐ *world*
☐ *electro*
☐ *pop / rock*
☐ *metal*
☐ *punk*
☐ *hip-hop / rap*
☐ *experimental*
☐ *other - give detail* ______

Have you regularly played an instrument (after 13 years of age, at least once per week, for at least 1 year)?

☐ *yes*
☐ *no*

If yes, which instrument? ______

On a scale from 0 to 6, how do you assess your own capacity to evaluate classical music (more particularly, pieces played on the violin)?

☐	☐	☐	☐	☐	☐	☐
0	1	2	3	4	5	6

0 = I am not competent at all -------------------- 6 = I am a connoisseur

Which criteria do you feel have influenced your vote?

What is your age category?

☐	☐	☐	☐	☐
15-18	*19-30*	*31-45*	*46-60*	*over 60*

What is your gender?

☐ *female* ☐ *male*

If you want, you can leave any comments here:

Thank you for participating in this music competition!

You can ***save*** *and close this document.*

2. SUPPLEMENTARY STATISTICS

2.1. Viewing order effect

Analysis of the full dataset with the generalized linear mixed-effects model (family = binomial) indicates that the viewing order affects participants' votes ($p < 0.001$; Table 1). In order to test the specific effect of the viewing order on participants' voting decisions, we analysed only data form the control condition—no moral information provided by the professor (teacher, n=32; high school, n=57; police, n = 55). We used a generalized linear mixed-effects models (family = binomial) with "viewing order" as the fixed factor, and class as a random effect.

Figure S1 shows that police and high school students preferred the second violinist seen ($p < 0.001$ and 0.004 respectively). In contrast, the teacher category was not significantly affected by the viewing order effect. This difference makes sense because preparatory schools for teachers provide more music training than police and high schools; thus, this category of participants was better at picking out the most talented violinist.

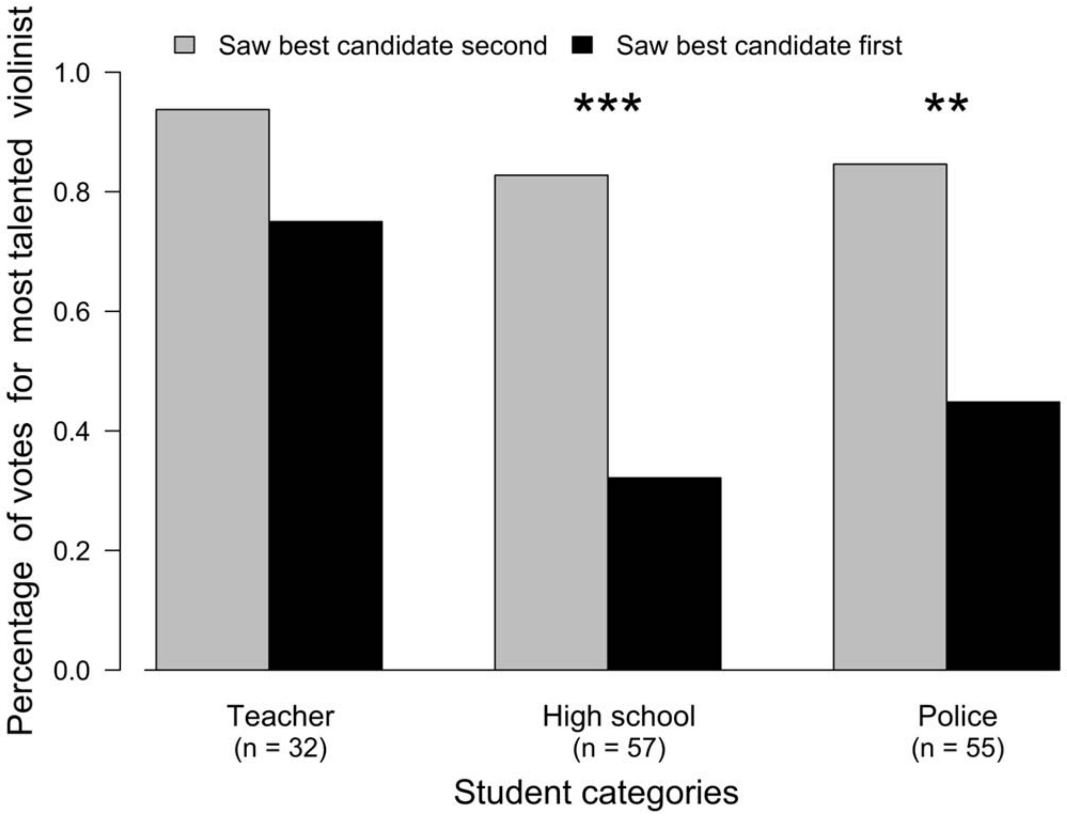

Figure S1. Percentage of votes for the most talented violinist among teacher, high school and police students when they saw the most talented violinist first (black bars) or second (grey bars). Significance levels: *** $p < 0.001$, ** $p = 0.004$, insignificant $p = 0.08$.

2.2. Correlation between viewing order and acknowledged influence of moral information

Among participants in the treatment condition (teacher, n=34; high school, n=16; police, n = 54), a modest proportion of police (19%) and high school (15%) students stated that moral information influenced their voting decision (Fig. 2). Although small, this proportion was greater than the 2.5% observed decrease in votes for the immoral violinist when she was described as immoral as opposed to socially integrated (Fig. 1). In order to explain this difference, we hypothesized that participants were more inclined to acknowledge receptivity to moral information if they would have voted against the most talented violinist anyway—even if she were described as socially integrated. Therefore, knowing that the viewing order effect biased participants' musical preference in favour of the second violinist seen (Fig S1), we expected to find a positive correlation between participants' acknowledged receptivity to moral information and viewing the most talented violinist first. We found (Fig. S2) such a correlation (one-tailed Fisher's exact test; $p = 0.025$), indicating that it is easier to respond to moral information when it points to a choice of action we are already inclined to make—i.e. acknowledge that the first violinist is immoral when we are already inclined to vote for the second.

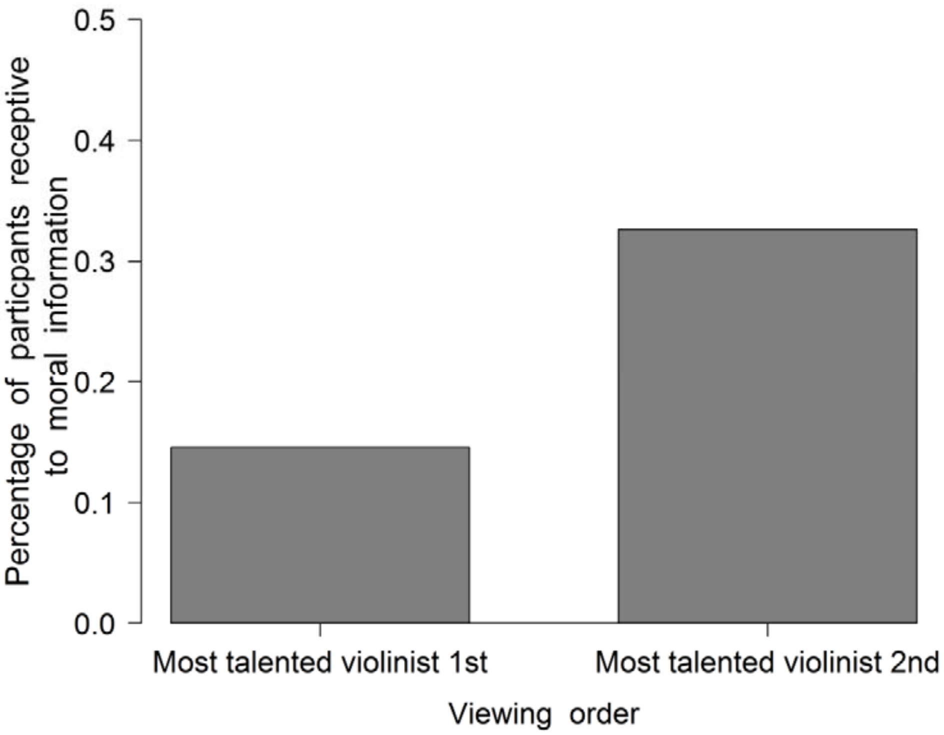

Figure S2. Percentage of participants who reported that moral information influenced their decision when they saw the most talented but immoral violinist first (left bar) or second (right bar).

2.3. Factors that were not significant

In addition to the data collected in the questionnaire (gender, interest in classical music, reported criteria influencing participants' votes), and the two factors randomized in all schools (moral information and violinist viewing order) we randomized three further factors in subsets of experiments.

(1) The content of the "technical" information provided by the professor on each violinist

Interviews of violinists' former music professor were composed of two types of pre-recorded strings; some containing "social information"—e.g. she is well integrated in her music class, or she has repetitively misbehaved—and other containing "technical information"—e.g. she usually is very right in her tone, or her play is full of colour. Descriptions about the violinists' technical abilities were necessary for the credibility of the cover story. For this, we used two pre-recorded interview stings with equivalent length and content. In order to insure that the difference in content did not impact on participants' votes, we randomized in most of our experiments the attribution of the two strings to each violinist (3 teacher classes, n=66; 3 high school classes, n=36; the large police class, n = 109). We used a generalized linear mixed-effects models (family = binomial) with "the content of technical information provided by the professor" as a fixed factor, and class as a random effect.

Table S1 shows that this factor had no significant effect on participants' votes.

Factor	Coefficient	z value	P
Content of technical information by professor (n = 211, 7 classes)	-0.297	-1.042	0.298

(2) The order in which the professor provided "social information" versus "technical information" on both violinists

To test whether participants are more influenced by moral information when it is provided last—that is, just before voting decision—we randomized in one high school (4 classes, n=86) the order in which the professor provided "social information" versus "technical information". We used a generalized linear mixed-effects model (family = binomial) with "the order of technical versus social information provided by the professor" as a fixed factor, and class as a random effect.

Table S2 shows that this factor had no significant effect on participants' votes.

Factor	Coefficient	z value	P
Order of technical versus social information by professor (n = 86, 4 classes)	-0.177	-0.405	0.685

(3) Which musician portrayed the most talented violinist

To ensure that participants did not prefer one musician over the other because of her physical characteristics or her attitude while playing, we randomized which musician portrayed the most talented violinist in the two high schools. In high school 1 experiment (n=86), we used Erzsebet's best and Maria-Grazia's worst pre-recorded performance, whereas in high school 2 experiment (n=37), we used Erzsebet's worst and Maria-Grazia's best pre-recorded performance. We used a generalized linear mixed-effects model (family = binomial) with "which musician portrayed the most talented violinist" as a fixed factor, and class as a random effect. Table S3 shows that this factor had no significant effect on participants' votes.

Table S3

**Factor**	**Coefficient**	**z value**	**P**
Which musician portrayed the most talented violinist (n = 122, 7 classes)	0.014	0.144	0.885
